# Balancing pH and yield: exploring itaconic acid production in *Ustilago cynodontis* from an economic perspective

**DOI:** 10.1186/s13068-024-02550-0

**Published:** 2024-07-17

**Authors:** Philipp Ernst, Katharina Maria Saur, Robert Kiefel, Paul-Joachim Niehoff, Ronja Weskott, Jochen Büchs, Andreas Jupke, Nick Wierckx

**Affiliations:** 1 Institute of Bio- and Geosciences IBG-1: Biotechnology, Forschungszentrum Jülich GmbH, Wilhelm-Johnen-Straße, 52428 Jülich, Germany; 2 Fluid Process Engineering (AVT.FVT), RWTH Aachen University, Forckenbeckstraße 51, 52074 Aachen, Germany; 3 Biochemical Engineering (AVT.BioVT), RWTH Aachen University, Forckenbeckstraße 51, 52074 Aachen, Germany

**Keywords:** *Ustilago cynodontis*, Itaconic acid, Low pH fermentations, Downstream processing, Techno-economic analysis

## Abstract

**Background:**

Itaconic acid is a promising bio-based building block for the synthesis of polymers, plastics, fibers and other materials. In recent years, *Ustilago cynodontis* has emerged as an additional itaconate producing non-conventional yeast, mainly due to its high acid tolerance, which significantly reduces saline waste coproduction during fermentation and downstream processing. As a result, this could likely improve the economic viability of the itaconic acid production process with Ustilaginaceae.

**Results:**

In this study, we characterized a previously engineered itaconate hyper-producing *Ustilago cynodontis* strain in controlled fed-batch fermentations to determine the minimal and optimal pH for itaconate production. Under optimal fermentation conditions, the hyper-producing strain can achieve the theoretical maximal itaconate yield during the production phase in a fermentation at pH 3.6, but at the expense of considerable base addition. Base consumption is strongly reduced at the pH of 2.8, but at cost of production yield, titer, and rate. A techno-economic analysis based on the entire process demonstrated that savings due to an additional decrease in pH control reagents and saline waste costs cannot compensate the yield loss observed at the highly acidic pH value 2.8.

**Conclusions:**

Overall, this work provides novel data regarding the balancing of yield, titer, and rate in the context of pH, thereby contributing to a better understanding of the itaconic acid production process with *Ustilago cynodontis*, especially from an economic perspective.

**Supplementary Information:**

The online version contains supplementary material available at 10.1186/s13068-024-02550-0.

## Background

Itaconic acid (ITA) is considered as a promising renewable building block for the synthesis of plastics, synthetic resins, fibers and other materials [1–4]. While itaconic acid and its derivatives also hold great potential in the medical and pharmaceutical sectors [5–7], a major opportunity exist in replacing acrylic acid and methacrylic acid in the polymer industry. However, this is only possible if the efficiency of the fermentation process can be increased to a point where it can compete with the petrochemical production [8, 9]. In view of the already high yield, high titer, high productivity of the industrially well-established itaconic acid production process with the filamentous fungus *Aspergillus terreus* DSM 23081 [10], the possibilities for further process improvements seem to be largely exhausted, requiring a qualitative breakthrough in other dimensions of the process window. Therefore, we focus on *Ustilago* since its stable yeast-like morphology may enable such further efficiency gains in scaled up fermentation processes by providing greater degrees of freedom in handling the fermentation broth [11, 12]. These gains may be further boosted by utilizing untreated industrial feedstocks [13], as the substrate cost remains a critical factor in itaconic acid production [14]. Moreover, the long history of safe use and lower biosafety level of *Ustilago* simplifies the itaconic acid production process in Europe compared to *A. terreus*.

In previous work, itaconate production with a deeply engineered *U. maydis* strain was optimized in terms of glucose feeding strategies [15]. A continuous fed-batch fermentation enabled itaconate production at 100% of the theoretical maximal yield during the production phase in a low-density fermentation. Although the acid tolerance of *U. maydis* is not very well investigated, it is generally considered to be less tolerant and therefore these fermentations were performed at a neutral pH. However, acidic pH values are beneficial for organic acid production [16, 17]. Therefore, we investigated *U.* *cynodontis*, an additional natural itaconate producer that has recently been engineered to higher efficiencies [18, 19]. The advantage of this species lies in the amalgamation of yeast-like morphology with high acid tolerance compared to *U. maydis* and *A. terreus*, thus enabling both easier handling and the utilization of the benefits from lower process pH values. A low pH value reduce bacterial growth, which generally minimizes the risk of contamination and may also allow to use a semi-sterile process, decreasing costs. However, a low pH is even more important for improving the efficiency of downstream processing (DSP) during the production of organic acids [2, 20]. Like other carboxylic acids, itaconic acid can be purified by various unit operations such as crystallization [2], extraction [21], adsorption, chromatography [22, 23], and membrane separation. Most of the itaconic acid purification techniques require the free acid. Therefore, the fully protonated state is required to obtain high yields during DSP [14, 21, 23–26]. As a result, the pH needs to be lowered after fermentation by acid addition. In the industrially used crystallization, this leads to the formation of a co-salt with the base from the fermentation. While for *A. terreus*, this is not an issue due to its low fermentation pH, the concentration and composition of the co-salt limits the yield and increases the cost of waste disposal for *U. maydis* [14, 27]. A techno-economic analysis already revealed that a slightly lower fermentation yield with *U.* *cynodontis* could be compensated by its low fermentation pH of 3.6 when compared to *U. maydis* [14]. According to previous research, it is known that *U. cynodontis* is capable of producing itaconate even at pH values below 3.6 [19]. Considering the pK_a_ values of itaconic acid (3.84 and 5.55), further reduction of the fermentation pH would shift itaconic acid dissociation towards the fully protonated species, significantly reducing the addition of pH control reagents and therefore costs. As a result, this could likely improve the economic viability of the itaconic acid production process with Ustilaginaceae.

A major factor in the establishment of *U. cynodontis* as a non-conventional itaconate producer was the deletion of *fuz7*, which arrested the cells in a yeast-like morphology thereby avoiding filamentous growth. Hosseinpour Tehrani, Saur et al. [19] determined the pH optimum for itaconate production at 3.6 with this morphology-engineered strain, but additional modifications were subsequently performed to enhance itaconate production. These genetic modifications include the deletion of the P450 monooxygenase encoding *cyp3*, the overexpression of the transcription regulator *ria1*, and the heterologous overexpression of the mitochondrial tricarboxylate transporter *mttA* from *A. terreus* [18]. These engineered strains were evaluated with a focus on downstream processing [14, 28, 29] and on different crude substrates like molasses [30], but typically only two pH values were used. Since the altered genetic constitution after metabolic engineering changed the product spectrum and interfered with the regulation of the itaconate gene cluster, the pH optimum for itaconate production with this optimized strain may have shifted. Given the importance of pH in fungal organic acid production we therefore systematically investigated the itaconate production capabilities of the new hyper-producing *U. cynodontis* strain in high resolution through controlled fed-batch fermentations. We explored the pH optimum of this strain in light of key performance indicators such as yield, titer, and rate, while also varying other aspects of the fermentation such as cell density. Additionally, the impact of pH on overall process economics was evaluated in a techno-economic analysis.

## Results and discussion

### Comparison of *U. maydis* K14 and *U. cynodontis* ITA MAX pH for itaconate production at low pH values

**Fig. 1:**
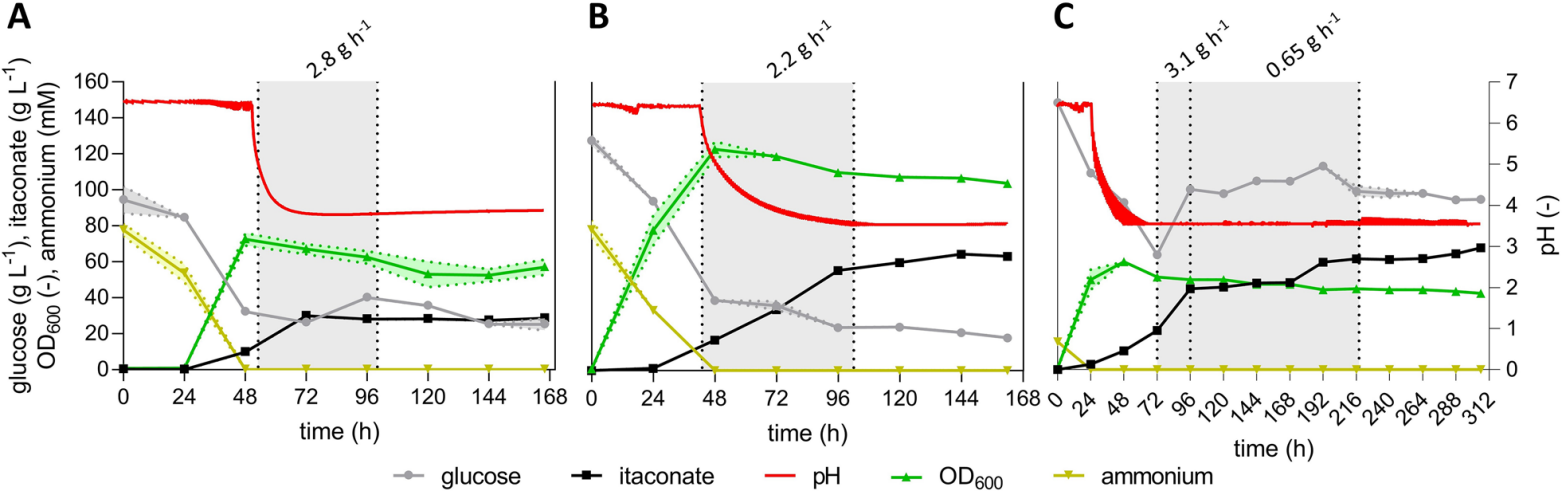
**Fed-batch fermentations with continuous feed of** ***U. maydis* K14 with high ammonium concentration (A) and** ***U. cynodontis* ITA MAX pH with high (B) and low (C) ammonium concentration**. Concentration of glucose (●), itaconate (■), pH (red line), OD_600_ (▲) and ammonium (▼) during fermentation in a bioreactor containing batch medium with approximately 120 g L^−1^ glucose and 75 mM NH_4_Cl. Vessels with a total volume of 2.3 L and a working volume of 1.0 L were used. The pH was controlled by automatic titration with 5 M NaOH. After the depletion of nitrogen (24 h and 48 h), the pH was allowed to drop from pH 6.5 to pH 3.6 through the production of itaconate. Cultures were fed with an additional 130 g glucose (50% w/v feeding solution) at a rate of 2.8 g h^−1^ for *U. maydis* K14 (53–100 h) and 2.2 g h^−1^ (43–102 h) or 0.65 g h^−1^ (96–218 h) for *U.* *cynodontis* ITA MAX pH. The feeding rates were estimated from glucose consumption rates of previous fermentations, aimed at keeping the glucose concentration at a relatively constant level of approximately 50 g L^−1^. The low-density cultures were overfed between 72 and 96 h (3.1 g h^−1^). The mean values with standard deviation of two independent biological replicates are shown

So far, fermentations of *U.* *maydis* MB215 ∆*cyp3* ∆MEL ∆UA ∆*dgat P*_*ria1*_::*P*_*etef*_ ∆*fuz7 P*_*etef*_*mttA*_K14, henceforth named strain K14 for ease of reference, were mainly performed at a neutral pH of 6.5 [15]. However, in shake flask cultivations, engineered *U. maydis* hyper-producers do not grow at acidic pH conditions, but still produce significant amounts of itaconate [31, 32]. Hence, itaconate production of *U. maydis* K14 was assessed in fed-batch fermentations with different pH values for growth and itaconate production phase (Fig. 1A). The remaining conditions such as a continuous glucose feed to avoid osmotic stress were similar to Becker et al., 2021 [15]. To gain a comprehensive comparison, *U.* *cynodontis* NBRC9727 ∆*fuz7* ∆*cyp3 P*_*etef*_*mttA P*_*ria1*_*ria1*, henceforth named strain ITA MAX pH for ease of reference, was cultured similarly under high-density (75 mM NH_4_Cl, Fig. 1B) and low-density conditions (15 mM NH_4_Cl, Fig. 1C). In previous studies, *U. maydis* K14 achieved higher product yields in the low-density fermentations [15].

Until 72 h, both high-density fermentations behaved similarly (Fig. 1A, B). The depletion of nitrogen was achieved and itaconate accumulated to approximately 30 g L^−1^. However, upon reaching lower pH levels, difference became apparent between the two species. In the case of you *U. maydis* K14, production almost completely stopped once the pH reached values below 4.0. On the contrary, *U.* *cynodontis* ITA MAX pH produced an additional 30 g L^−1^ itaconate after reaching acidic pH values. Remarkably, the *U. maydis* K14 culture did not even reach the final pH value 3.6, although the glucose concentration still declined until the end of the fermentation. This continued glucose uptake in the absence of further production indicates a very high metabolic energy demand for maintaining intracellular pH homeostasis. These results show that *U. maydis* K14 is not suitable to produce itaconate at lower pH values and is possibly more sensitive towards weak acid stress. Consequently, *U. cynodontis* ITA MAX pH was identified as a preferable candidate for subsequent characterization. If itaconic acid is to become a bulk chemical, yield is one of the most relevant production parameter because substrate cost is a decisive price-determining factor [14]. The availability of nitrogen and the resulting C/N ratio offer a dimension to optimize the product to substrate yield by controlling the biomass density. Typically, lower nitrogen levels result in higher yields but also lower productivities [33]. In previous fed-batch fermentations of *U. maydis* K14 with a reduced ammonium concentration, itaconate was produced at the maximal theoretical yield of 0.72 ± 0.02 g_ITA_ g_GLC_^−1^ during the production phase [15]. A similar trend was observed for *U. cynodontis* ITA MAX pH during the low-density fermentation (Fig. 1C). This fermentation resulted in a similar itaconate titer of 67.8 ± 0.7 g L^−1^ compared to the high-density fermentation. Interestingly, the fivefold reduction in ammonium chloride as growth-limiting nutrient only resulted in an approximately twofold reduction of the maximum OD_600_ value as well as of the overall production rate (0.22 ± 0.01 g L^−1^ h^−1^). A similar phenomenon was observed for *U. maydis* [15]. The lower substrate requirement for biomass production enabled a higher yield of 0.55 ± 0.02 g_ITA_ g_GLC_^−1^. When disregarding the glucose consumed during the first 24 h in the growth phase, this fermentation achieved the theoretical maximal yield of 0.72 ± 0.01 g_ITA_ g_GLC_^−1^. This yield is the highest yield ever reported for *U. cynodontis*. Compared to previously published low-density pulsed fed-batch fermentation with a pH shift from 6.0 to 3.6, the fed-batch with continuous feed increased the titer by 62% and the yield by 41%, while the overall productivity remained nearly constant. These results clearly illustrate the benefit of a continuous glucose feed, preventing osmotic shocks caused by pulsed feeding. However, it is to note that the glucose concentration during feeding was significantly higher than in the pulsed fed-batch fermentation. Although a higher osmotic stress due to elevated glucose concentration would be expected, it is also plausible that approximately 100 g L^−1^ glucose represents a threshold concentration for achieving more efficient itaconate production while maintaining relatively low osmotic stress. These findings are in line with those reported for itaconate production with *A. terreus*, where the highest yields were obtained at glucose concentration between 120 and 200 g L^−1^ [34]. Almost the same is reported for citrate production in *A.* *niger* [35]. This phenomenon should be further investigated for itaconate production with Ustilaginaceae.

### Comparison of the itaconate production capabilities of *U. cynodontis* ITA MAX pH at neutral and acidic pH values

Previous research has demonstrated that *U. cynodontis* is also able to grow at the acidic pH value 3.6 [19]. To investigate the impact of reduced pH values throughout the entire fermentation process, additional fed-batch fermentations were conducted as described above. Biomass formation, substrate consumption (glucose and ammonium) and itaconic acid production capabilities achieved at pH 3.6 were compared to values obtained from fermentations at pH 6.5.

**Fig. 2:**
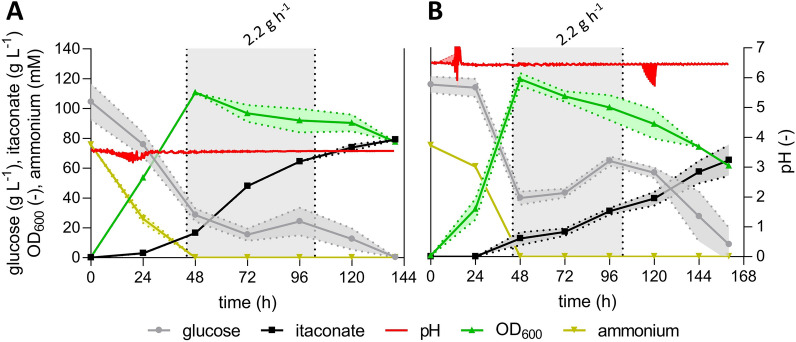
**High-density fed-batch fermentations of** ***U. cynodontis*** **ITA MAX pH with continuous feed at pH 3.6 (A) and pH 6.5 (B)**. (**A**, **B**) concentration of glucose (●), itaconate (■), pH (red line), OD_600_ (▲) and ammonium (▼) during fermentation in a bioreactor containing batch medium with approximately 110 g L^−1^ glucose and 75 mM NH_4_Cl. Vessels with a total volume of 2.3 L and a working volume of 1.0 L were used. The pH was controlled by automatic titration with 5 M NaOH. Cultures were fed with an additional 130 g glucose (50% w/v feeding solution) at a rate of 2.2 g h^−1^ (44–103 h). The feeding rates were estimated from glucose consumption rates of previous fermentations, aimed at keeping the glucose concentration at a constant level of approximately 50 g L^−1^. The mean values with standard deviation of two independent biological replicates are shown

In the neutral pH fermentation, 64.7 ± 10.5 g L^−1^ itaconate was produced within approximately 160 h with an overall productivity of 0.40 ± 0.06 g L^−1^ h^−1^ and the yield 0.42 ± 0.02 g_ITA_ g_GLC_^−1^ (Fig. 2B). Previous fed-batch fermentation with a pH-shift from 6.5 to 3.6 achieved similar key performance indicators (KPIs) (Fig. 1B). However, the low pH fermentation resulted in a higher itaconate yield of 0.49 ± 0.01 g_ITA_ g_GLC_^−1^ (Fig. 2A). In addition, the overall productivity was increased by 39% (0.57 ± 0.01 g L^−1^ h^−1^) and the titer by 22% (79.2 ± 1.3 g L^−1^). These results show that *U. cynodontis* ITA MAX pH not only tolerates acidic conditions, it actually produces better at lower pH values. This result is promising, given that the acidic pH fermentation required approximately 3.5-fold less NaOH compared to the neutral pH fermentation (112 mL and 398 mL 5 M NaOH solution). The KPIs are in good accordance with those previously achieved with this strain using a constant glucose feed controlled by an inline glucose sensor (78.6 g L^−1^, 0.45 g_ITA_ g_GLC_^−1^, 0.42 g L^−1^ h^−1^) [19]. Less optimal progenitor strains of *U. cynodontis* ITA MAX pH are capable of producing itaconate even at pH levels below 3.6 [19]. Given the pK_a_ values of itaconic acid of 3.84 and 5.55, further reduction of the fermentation pH is expected to still significantly reduce base consumption [36]. Thereby, costs associated with pH adjusting reagents can be further reduced. In addition, less hydrochloric acid (HCl) is necessary for DSP. This leads to a lower amount of co-salt in the fermentation broth, which can, therefore, be further concentrated which increases the crystallization yields. As shown by Saur et al. [14], this increased yield in DSP is able to compensate for partial yield loss in fermentation (cf. introduction). As a result, this capability holds the potential to improve the economic viability of the itaconic acid production process with *U. cynodontis*.

### Identification of the pH optimum for itaconate production with *U. cynodontis* ITA MAX pH

**Fig. 3:**
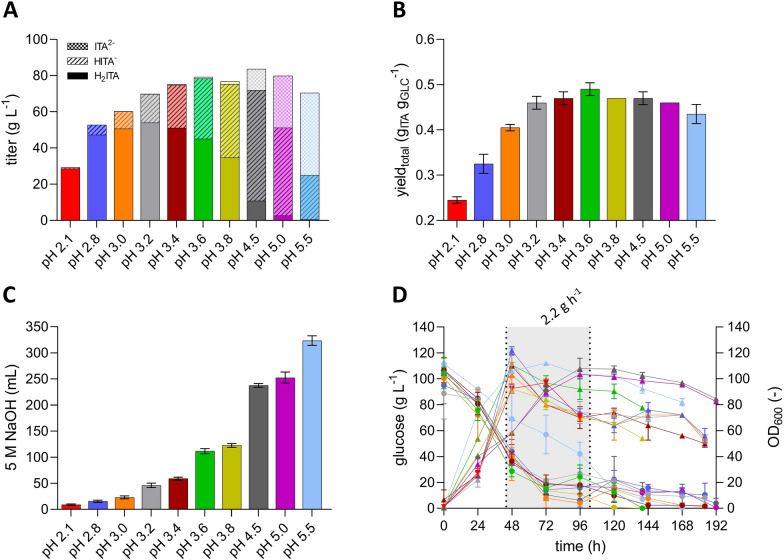
**Controlled fermentations of** ***U. cynodontis* ITA MAX pH at different process pH values**. (**A**) distribution of protonation states of itaconic acid, (**B**) yield_total_, (**C**) added volumes of 5 M NaOH and (**D**) concentration of glucose (●) and OD_600_ (▲) during fermentation at different pH values in bioreactors containing batch medium with approximately 110 g L^−1^ glucose and 75 mM NH_4_Cl. Vessels with a total volume of 2.3 L and a working volume of 1.0 L were used. The colors in Figure D indicate fermentations at the pH values as shown in the panels A-C. The pH was controlled by automatic titration with 5 M NaOH. After the depletion of nitrogen (48 h), the pH was allowed to naturally drop to the corresponding lower pH value. pH values above 3.6 were manually adjusted with 5 M NaOH. pH 2.1 represents the lowest possible pH value that can be achieved with *U.* *cynodontis* ITA MAX pH. This pH value was determined in a high-density batch fermentation with approximately 200 g L^−1^ glucose and an uncontrolled pH value during the production phase. All other cultures were fed with an additional 130 g glucose (50% w/v feeding solution) at a rate of 2.2 g h^−1^ (44–103 h). The mean values with standard deviation of two independent biological replicates are shown

The pH plays a crucial role as it determines the itaconic acid species (H_2_ITA, HITA^−^, ITA^2−^) distribution during the fermentation process. The species can be derived from the pH values using the Henderson-Hasselbalch equation and are shown in Fig. 3A. On one hand, the protonated acid greatly facilitates DSP as it avoids additional acid use and salt coproduction as described above. On the other hand, fully protonated itaconic acid negatively impacts the efficiency of the fermentation as it leads to weak acid uncoupling, which increases the maintenance demand through energy-driven export of protons, and it possibly increases product inhibition by raising the intracellular itaconate concentration [36] Therefore, it is crucial to carefully determine the optimum pH value in order to balance these counteracting effects and achieve the overall most cost-efficient itaconate production. However, the pH optimum for itaconate production with *U. cynodontis* has so far only been determined with the sub-optimal production strain containing only the *fuz7* deletion. To examine the pH optimum of the new itaconate hyper-producing strain ITA MAX pH, a series of pH controlled fed-batch fermentations were conducted in standardized conditions. To avoid growth defects due to pH values below 3.6, the initial biomass production phase was performed at pH 3.6 for all fermentations. Following the depletion of the nitrogen source, the pH was allowed to drop to the corresponding lower pH value. To adjust the pH value above values of 3.6, NaOH was added.

During the initial biomass production phase, the reached optical density was similar across all experiments. However, after the pH was adjusted to the corresponding value being tested, differences in the cell densities became apparent. pH values below 3.6 resulted in decreased optical densities (Fig. 3D), indicating that cells were stressed by the produced acid. The stress is most likely caused by weak acid uncoupling, which is more prominent at low pH values due to higher fractions of the double-protonated species (Fig. 3A). The increased weak acid uncoupling at these lower pH values is also reflected in reduced yields (Fig. 3B). The lowest yield was observed at the minimal pH value of 2.1, which was determined in a batch fermentation without pH control during the itaconate production phase between 48 and 96 h. However, the NaOH consumption during this fermentation was 36-fold reduced compared to the fermentation at pH 5.5 (Fig. 3C, Additional file 1). The highest yield with moderate base addition was achieved at pH 3.6, similar to what was previously determined for the morphology-engineered strain. The double-protonated form remained relatively constant within the pH range of 2.8 and 3.4, suggesting a potential inhibitory threshold for the cells (Fig. 3A). At pH 3.6, there is a significant reduction in H_2_ITA. This reduction could explain the high KPIs observed at this pH value, indicating that H_2_ITA concentrations are key to achieving maximum KPIs. Considering only the yield, the additional genetic modification did not change the pH optimum. The morphology-engineered strain also achieved the highest itaconate titer at 3.6, and showed declining titers with increasing pH values. Regarding itaconate titers, it seemed that higher pH values negatively impact the regulation of the itaconate cluster genes of the *fuz7* variant [19]. However, the new itaconate hyper-producing strain showed increasing titers with increasing pH values until a pH of 5.0 (Fig. 3A). The overexpression of *ria1* may have contributed to an increased tolerance of the strain towards higher product concentrations. It may also be possible that the overexpression of *ria1* reduced the pH dependency of the regulation of the itaconate cluster genes. One of the main benefits of production at more neutral pH values lies in the potentially higher titers [10, 36].

In order to determine the maximum itaconate titer that can be obtained with this strain at pH 5.0, an additional fed-batch fermentation was performed with a prolonged feeding phase. We selected pH 5.0, because the ITA titer increased linearly until the end of the cultivation. At pH 4.5, the ITA titer flattened out.

**Fig. 4:**
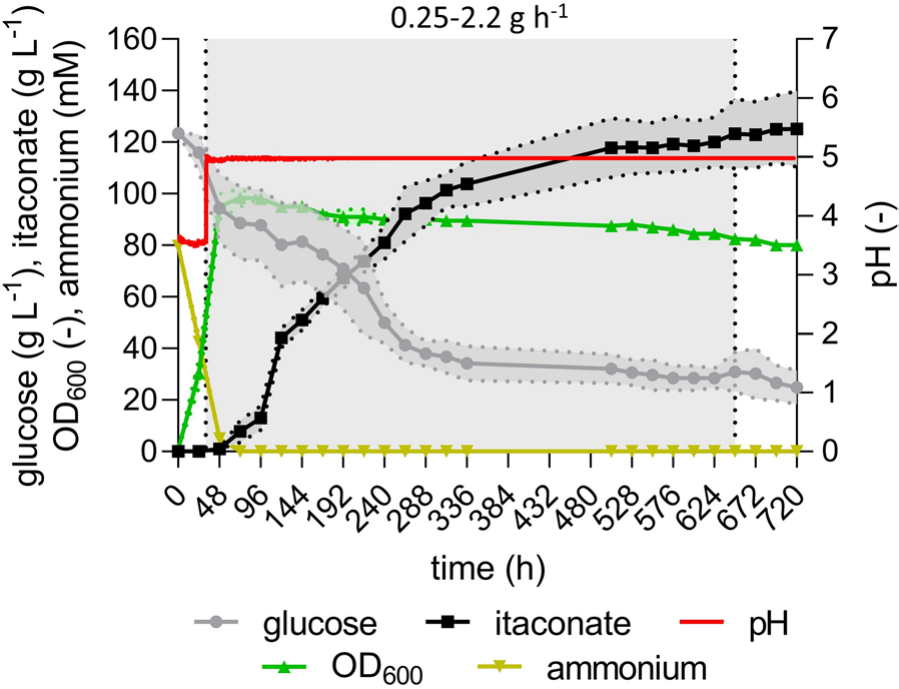
**High-density fed-batch fermentation at pH 5.0 with a prolonged feeding phase of** ***U. cynodontis* ITA MAX pH**. Concentration of glucose (●), itaconate (■), pH (red line), OD_600_ (▲) and ammonium (▼) during fermentation in a bioreactor containing batch medium with approximately 120 g L^−1^ glucose and 75 mM NH_4_Cl. Vessels with a total volume of 2.3 L and a working volume of 1.0 L were used. The pH was controlled by automatic titration with 5 M NaOH. After approximately 48 h, the pH was adjusted to pH 5 and afterwards maintained at this value until the end of the fermentation. Cultures were fed with an additional 540 g glucose (70% w/v feeding solution) at rates between 0.25 and 2.2 g h^−1^ (32–648 h). The feeding profile is shown in Additional file 2. The feeding rates were manually adjusted in order to maintain the glucose concentration at a constant level of approximately 50 g L^−1^. The mean values with standard deviation of two independent biological replicates are shown

In the fed-batch fermentation with a prolonged feeding phase, the itaconate titer kept linearly increasing up to 264 h up to approximately 92.3 ± 10.7 g L^−1^ at a rate of 0.34 ± 0.01 g L^−1^ h^−1^ and a yield of 0.42 ± 0.01 g_ITA_ g_GLC_^−1^ (Fig. 4). During the remaining fermentation time, a linear increase of the itaconate titer could still be observed, however, with a strongly reduced rate as the product inhibition became more and more prominent, taking another 434 h to produce only 32.9 ± 4.0 g L^−1^ additional itaconate. This fermentation reached a final itaconate titer of 125.2 ± 14.6 g L^−1^, one of the highest itaconate titers reported for Ustilaginaceae with NaOH titration. The itaconate concentration remained constant between 696 and 720 h, indicating that the maximum titer was finally reached after approximately one month of fermentation time. In total, this fermentation resulted in an overall productivity of 0.17 ± 0.02 g L^−1^ h^−1^ and a yield of 0.36 ± 0.01 g_ITA_ g_GLC_^−1^. Although a very high titer could be achieved through extended feeding, this came at the major expense of a lower yield end rate. Despite the reduced weak acid stress at the pH value of 5.0 and the higher itaconate production per cell, this fermentation highlighted the strong inhibitory effect of elevated product titers on the overall KPIs, indicating a major efficiency loss at titers above 92.3 ± 10.7 g L^−1^ even at a higher pH value. This result highlights that not only elevated H_2_ITA concentrations are inhibitory for the cells, but also higher overall product titers most probably due to increased osmotic stress.

In summary, the engineered *U. cynodontis* ITA MAX pH strain shows an extended operational range in term of pH for itaconate production compared to the previous morphology-engineered strain, particularly in terms of itaconate titers. The carbon balances of all fermentations are shown in Additional file 3. Analogous to the morphology-engineered strain, the achieved KPIs for the hyper-producing strain exhibited a moderate decrease at pH values below 3.6, which became more prominent at pH values below 3.0 (Fig. 3B). However, the volumes of NaOH added during these low-pH fermentations were also significantly reduced (Fig. 3C) and associated reductions in acid consumption and saline waste production during DSP can be expected. To assess whether these can economically compensate for the losses in fermentation yield at pH values lower than 3.6, an operational cost analysis was performed. We also added the economic results for fed-batch fermentations conducted at higher pH values than 3.6 to provide a full picture of the cost structure changes.

### Identification of the pH optimum by operative cost analysis

**Fig. 5:**
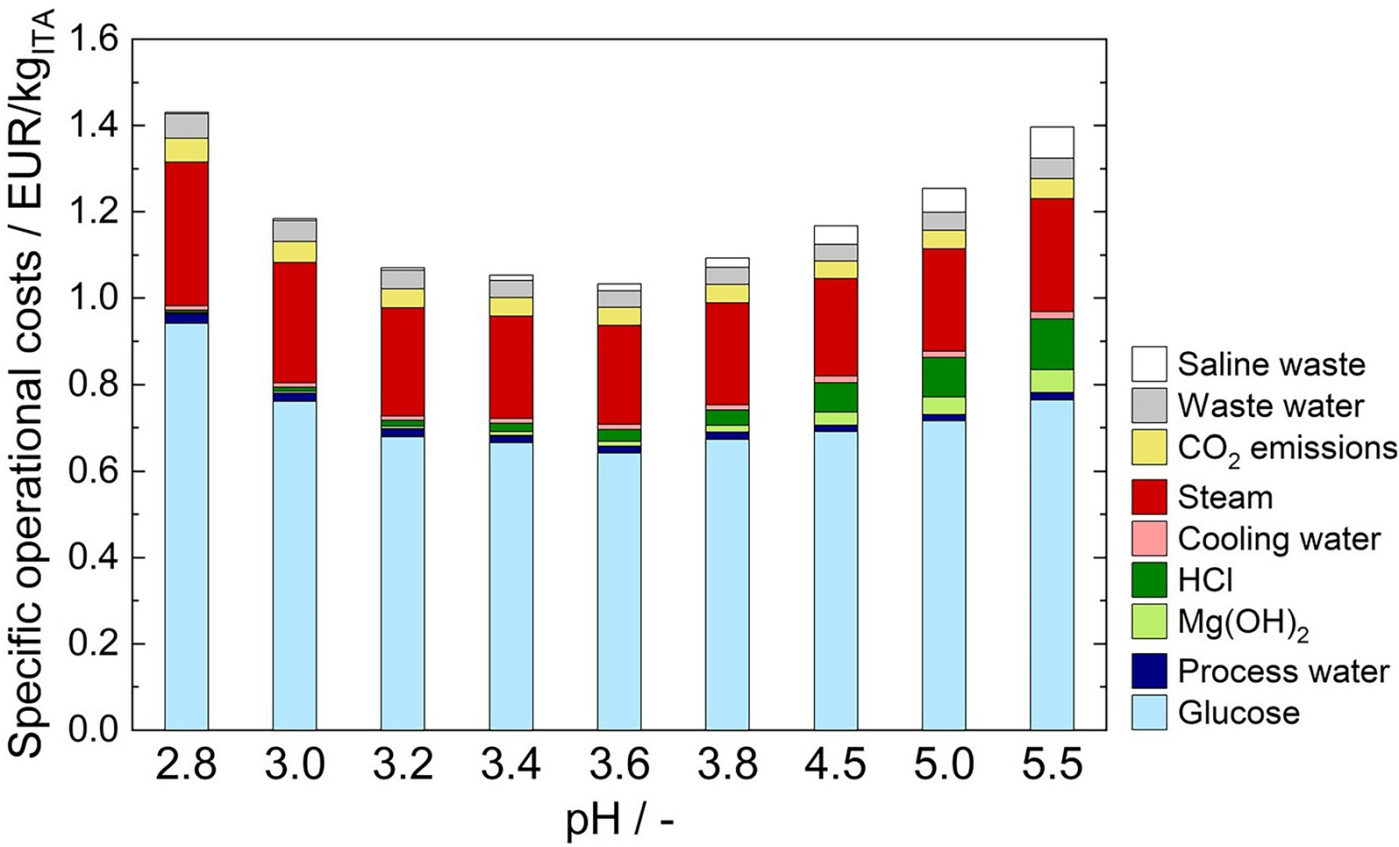
Specific operational costs for fed-batch fermentations at different pH values

The process KPIs from the fed-batch fermentations served as an input parameter for the simulation. Those consist of the product titer and the fermentation yield. The pH value during the product formation phase was also considered (Fig. 3A, B). The results of the cost analysis are displayed in Fig. 5.

As expected, the simulated costs associated to acid and base use and saline waste disposal decrease with lower fermentation pH. Reduced amounts of HCl and saline waste could also be confirmed in crystallization experiments using real cultivation supernatants from batch fermentations (Additional file 4). However, those costs comprise only a small fraction of the total operational costs. Independent of the selected pH, the total operational costs are most strongly influenced by overall substrate yield. The outstanding fermentation yield at pH 3.6 cuts costs significantly while specific operational costs are visibly larger at high and low pH values due to the poor substrate to product conversion. This cannot be outweighed by higher DSP yields at lower pH as discussed in Saur et al. [14]. The specific operational costs of approximately 1.04 EUR kg^−1^ at pH 3.6 achieved in this work are approximately 0.40 EUR kg^−1^ lower than those previously obtained for this strain [14]. Nevertheless, to successfully compete with the petrochemical production of the counterparts acrylic acid and methacrylic acid, it is necessary to further reduce the production costs [37].

## Conclusion

From an economic perspective, the process pH should not be controlled below 3.6 during itaconic acid production as the reduced amounts of salt waste production cannot counterbalance the yield deficits. However, lower pH values during the production phase may still offer advantages. Due to the further reduced risk of process contamination, semi-sterile industrial feedstocks might be fed during the production phase thereby reducing costs. The general feasibility to produce itaconate at the highly acidic pH value 2.8 using the side stream thick juice from the sugar industry could already be successfully demonstrated (Additional file 5). In addition, in situ product removal is greatly facilitated by direct production of the protonated itaconic acid at these lower pH values [21, 28]. Therefore, the low fermentation pH of 2.8 is expected to significantly enhance product removal efficiency, which may further reduce the DSP costs. These effects should be further investigated. Future studies could also comprise a techno-economic analysis excluding the initial biomass production phase since Hosseinpour Tehrani, Saur et al. [19] showed that *U. cynodontis* ITA MAX pH can be subjected to repeated batch fermentations. In this way, the same biomass could be used multiple times for itaconate production, potentially leading to a more improved cost analysis of the itaconate production process with Ustilaginaceae.

Overall, this study provides exquisite data regarding the production strain properties and production capabilities, potentially enabling the development and implementation of an even more cost-effective itaconic acid production process with Ustilaginaceae in the future.

## Materials and methods

### Chemicals and strains

All chemicals used in this study were obtained from Sigma-Aldrich (St. Louis, USA), Thermo Fisher Scientific (Waltham, USA), or VWR (Radnor, USA) and were of analytical grade. Thick juice was supplied by Pfeifer & Langen Industrie- und Handels-KG.

The strain *U. cynodontis* NBRC9727 ∆*fuz7* ∆*cyp3 P*_*etef*_*mttA P*_*ria1*_*ria1* [18] and the strain *U. maydis* MB215 ∆*cyp3* ∆MEL ∆UA ∆*dgat P*_*ria1*_::*P*_*etef*_ ∆*fuz7 P*_*etef*_*mttA*_K14 [15] were used in this study.

### Bioreactor cultivations

Controlled fed-batch cultivations on glucose were performed in a DASGIP® Bioblock (Eppendorf, Germany). The process was controlled using the Eppendorf DASware® control software (Eppendorf, Germany). Vessels with a total volume of 2.3 L and a working volume of 1.0 L were used. Batch cultivations on thick juice were performed in a Sartorius fermenter controlled by the BioPAT MFCS software. Vessels with a total volume of 2.0 L and a working volume of 1.0 L were used. All cultivations were performed in batch medium according to Geiser et al. [38] containing 0.2 g L^−1^ MgSO_4_·7H_2_O, 0.01 g L^−1^ FeSO_4_·7H_2_O, 0.5 g L^−1^ KH_2_PO_4_, 1 g L^−1^ yeast extract (Merck Millipore, Germany), 1 mL L^−1^ vitamin solution, 1 ml L^−1^ trace element solution and varying concentrations of glucose, thick juice, and NH_4_Cl, as indicated. The vitamin solution contained (per liter) 0.05 g d-biotin, 1 g d-calcium pantothenate, 1 g nicotinic acid, 25 g myo-inositol, 1 g thiamine hydrochloride, 1 g pyridoxol hydrochloride, and 0.2 g para-aminobenzoic acid. The trace element solution contained (per liter) 1.5 g EDTA, 0.45 g of ZnSO_4_·7H_2_O, 0.10 g of MnCl_2_·4H_2_O, 0.03 g of CoCl_2_·6H_2_O, 0.03 g of CuSO_4_·5H_2_O, 0.04 g of Na_2_MoO_4_·2H_2_O, 0.45 g of CaCl_2_·2H_2_O, 0.3 g of FeSO_4_·7H_2_O, 0.10 g of H_3_BO_3_ and 0.01 g of KI. During cultivation, the pH was kept constant at the corresponding value by automatic addition of 5 M NaOH or 1 M HCl. The DO was controlled at 30% by a cascade mode: first agitation 800–1200 rpm (0–40% DOT controller output); second air flow 1–2 vvm (40–80% DOT controller output); third oxygen 21–100% oxygen (80–100% DOT controller output). The cultivation was performed at 30 °C. The bioreactor was inoculated to a final OD_600_ of 0.75 from an overnight pre-culture grown in screening medium according to Geiser et al. [38] containing 50 g L^−1^ glucose and 100 mM MES buffer. 0.5 mL Antifoam 204 (Sigma, A6426) was added in the beginning of the cultivation and afterwards every 24 h.

### Analytical methods

Identification and quantification of products and substrates in the supernatants was performed using a High Performance Liquid Chromatography (HPLC) 1260 Infinity system (Agilent, Waldbronn, Germany) with an ISERA Metab AAC column 300 × 7.8 mm column (ISERA, Germany). Separation was achieved by using an isocratic elution program at a flow rate of 0.6 mL min^−1^ and a temperature of 40 °C with 5 mM sulfuric acid as a solvent. For detection, a diode array detector (DAD) at 210 nm and a refraction index (RI) detector was used. All samples were filtered with Rotilabo® syringe filters (CA, 0.20 μm, Ø 15 mm) and afterwards diluted with ddH_2_O. Analytes were identified via retention time compared to corresponding standards. Data analysis was performed using the Agilent OpenLAB Data Analysis—Build 2.200.0.528 software (Agilent, Waldbronn, Germany). The ammonium concentration in culture samples was determined using the colorimetric method after Willis et al. [39]. 10 µL culture supernatant was combined with 200 µL reagent (8 g Na-Salicylate, 10 g Trisodiumphosphate, 0.125 g Na-Nitroprusside), followed by the quick addition of 50 µL hypochlorite solution. After color development occurred (at least 15 min at RT), absorbance at 685 nm was measured in a flat-bottomed MTP plate without lid using a spectophometer. Ammonium concentrations were calculated using a standard curve of ammonium. Cell densities were quantified by optical density measurement at 600 nm wavelength (OD_600_) by use of cuvettes and a spectrophotometer. Samples were diluted appropriately with the respective medium to fall within the linear measuring range of the photometer between absolute values of 0.2 and 0.4. For DCW determination, 2 mL culture broth was centrifuged at maximum speed followed by drying the pellet for 48 h at 65 °C and afterwards weighing it.

### Process design and operative cost analysis

The presented cost analysis is performed based on the process design described by Saur et al. [14] for itaconic acid production with *Ustilago* species. The according flowsheet is illustrated by a block flow diagram in Additional file 6.

The fermenter is fed with a diluted glucose feed of 500 g L^−1^. During itaconic acid formation, the pH is maintained by base addition. The broth is separated from the cells by sterile filtration. Afterward, the filtered broth is concentrated by evaporation up to an itaconic acid concentration of 350 g L^−1^. The pH is then lowered by acid addition so that a pH of approximately 2.8 is attained after crystallization. In the cooling crystallizer, the temperature is decreased to 15 °C at atmospheric pressure. To increase the itaconic acid yield in the DSP, the purification sequence is repeated. Water is further removed from the mother liquor by a second evaporator up to a concentration at which a co-crystallization of itaconic acid and inorganic salt in a second cooling crystallizer can just be avoided. The inorganic salt-containing liquid stream is subsequently disposed. However, the itaconic acid solid fractions are dissolved in water at 80 °C to remove residual contaminants and increase the purity of the final itaconic acid crystals. The elevated temperature requires only moderate amounts of water for dilution and avoids large heat requirements for evaporation in succeeding process steps. To decolorize the dissolved itaconic acid stream, an activated carbon treatment is performed. Finally, the solution is fed to an evaporative crystallizer. The mother liquor is recycled and mixed with the filtered fermentation broth while the itaconic acid crystals are fed to a dryer.

The process design requires the use of Mg(OH)_2_ as base in the fermenter. The subsequent acidic pH shift in the DSP is performed with HCl to form the highly soluble co-salt MgCl_2_. To alleviate the experimental investigation, fed-batch fermentations conducted for this work are pH-controlled by 5 M NaOH solution instead of a Mg(OH)_2_ suspension, which easily causes blocking of small-diameter tubing.

The flowsheet is modeled using Aspen Plus (V11) (Aspen Technology, Inc., Bedford, MA, USA). Calculated material streams and energy demands are used for the cost analysis. The details of the modeling framework and pricing are outlined in Saur et al. [14].

### Supplementary Information

Additional file 1: Scatter plot of yield versus volumes of 5 M NaOH. Additional file 2: Feeding profile with 70 % w/v glucose solution during high-density fed-batch fermentation with a prolonged feeding phase of *U. cynodontis* ITA MAX pH. Additional file 3: Carbon balances of all fermentations showing C-atoms in % of ITA, erythritol (ERY), CO2, and DCW. Additional file 4: Crystallization of itaconate from batch fermentations conducted at pH 3.6 and pH 2.8. Additional file 5: High-density batch fermentation of *U. cynodontis* ITA MAX pH with thick juice as a sole carbon source. Additional file 6: Block flow diagram of multiple crystallization process (simplified from Saur et al. 2023 (14)).

## Data Availability

All data generated or analysed during this study are included in this published article and its supplementary information files.

## Abbreviations

ITA: Itaconic acid
MTM: Modified Tabuchi medium
YEPS: Yeast extract, peptone, sucrose medium
HPLC: High-performance liquid chromatography
DSP: Downstream processing
ISPR: In situ product removal
OD: Optical density
DCW: Dry cell weight
NaOH: Sodium hydroxide
MES: 2-(N-morpholino) ethane sulfonic acid
KPIs: Key performance indicators
