# Supplementary material for: Balancing pH and yield: exploring itaconic acid production in Ustilago cynodontis from an economic perspective
Source: Biotechnol Biofuels Bioprod. 2024 Jul 17;17:103. doi: 10.1186/s13068-024-02550-0 (PMC11253337; doi:10.1186/s13068-024-02550-0)
Supplement: Supplementary file 1 — Additional file 1: Scatter plot of yield versus volumes of 5 M NaOH. Additional file 2: Feeding profile with 70 % w/v glucose solution during high-density fed-batch fermentation with a prolonged feeding phase of U. cynodontis ITA MAX pH. Additional file 3: Carbon balances of all fermentations showing C-atoms in % of ITA, erythritol (ERY), CO2, and DCW. Additional file 4: Crystallization of itaconate from batch fermentations conducted at pH 3.6 and pH 2.8. Additional file 5: High-density batch fermentation of U. cynodontis ITA MAX pH with thick juice as a sole carbon source. Additional file 6: Block flow diagram of multiple crystallization process (simplified from Saur et al. 2023 (14)). [file 13068_2024_2550_MOESM1_ESM.docx]

**Additional files to the manuscript:**

**Balancing pH and Yield: Exploring Itaconic Acid Production in *Ustilago cynodontis* from an Economic Perspective**

Philipp Ernst^1^, Katharina Maria Saur^2^, Robert Kiefel^2^, Paul-Joachim Niehoff^3^, Ronja Weskott^1^, Jochen Büchs^3^, Andreas Jupke^2^, Nick Wierckx^*1^

^1^Institute of Bio- and Geosciences IBG-1: Biotechnology, Forschungszentrum Jülich GmbH, Wilhelm-Johnen-Straße, 52428 Jülich, Germany

^2^Fluid Process Engineering (AVT.FVT), RWTH Aachen University, Forckenbeckstraße 51, 52074 Aachen, Germany

^3^Biochemical Engineering (AVT.BioVT), RWTH Aachen University, Forckenbeckstraße 51, 52074 Aachen, Germany

*Corresponding author: [n.wierckx@fz-juelich.de](mailto:n.wierckx@fz-juelich.de)


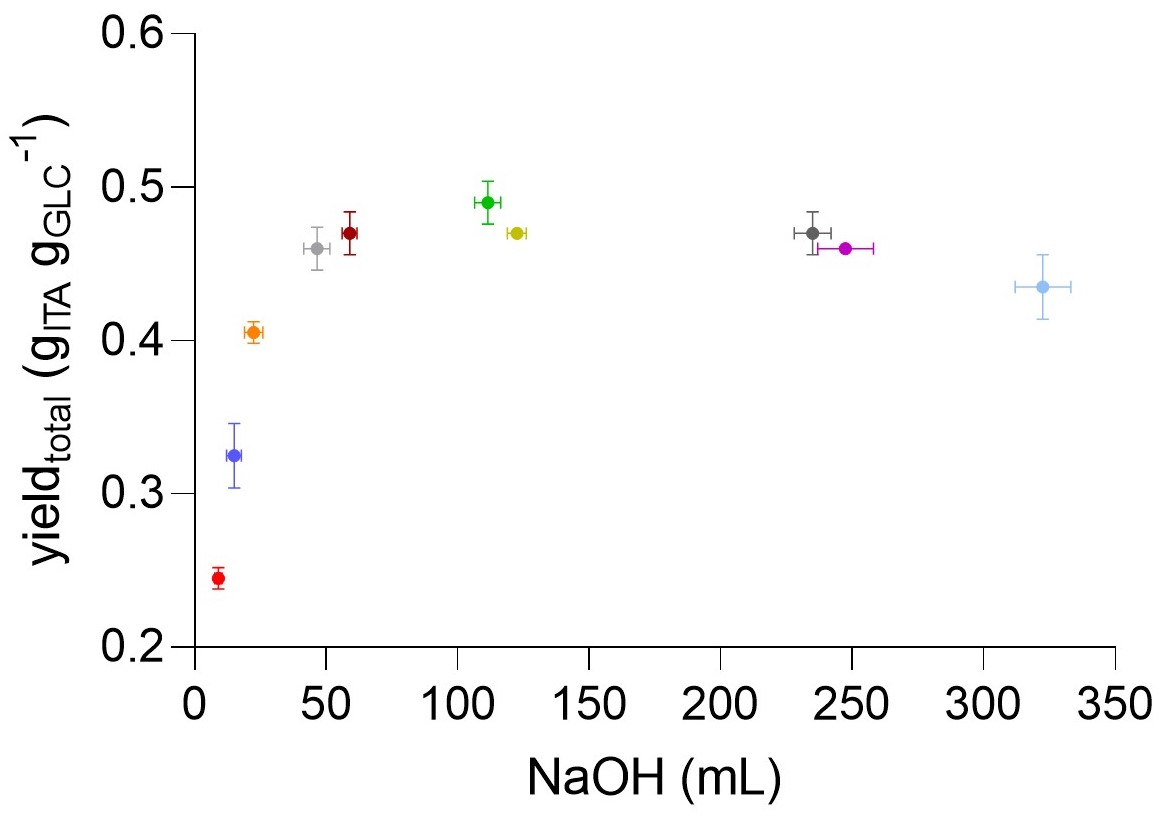


**Additional file 1: Scatter plot of yield versus volumes of 5 M NaOH.**

The colors indicate fermentations at the pH values as shown in figure 3 A-C.

**Additional file 2: Feeding profile with 70 % w/v glucose solution during high-density fed-batch fermentation with a prolonged feeding phase of *U. cynodontis* ITA MAX pH.**


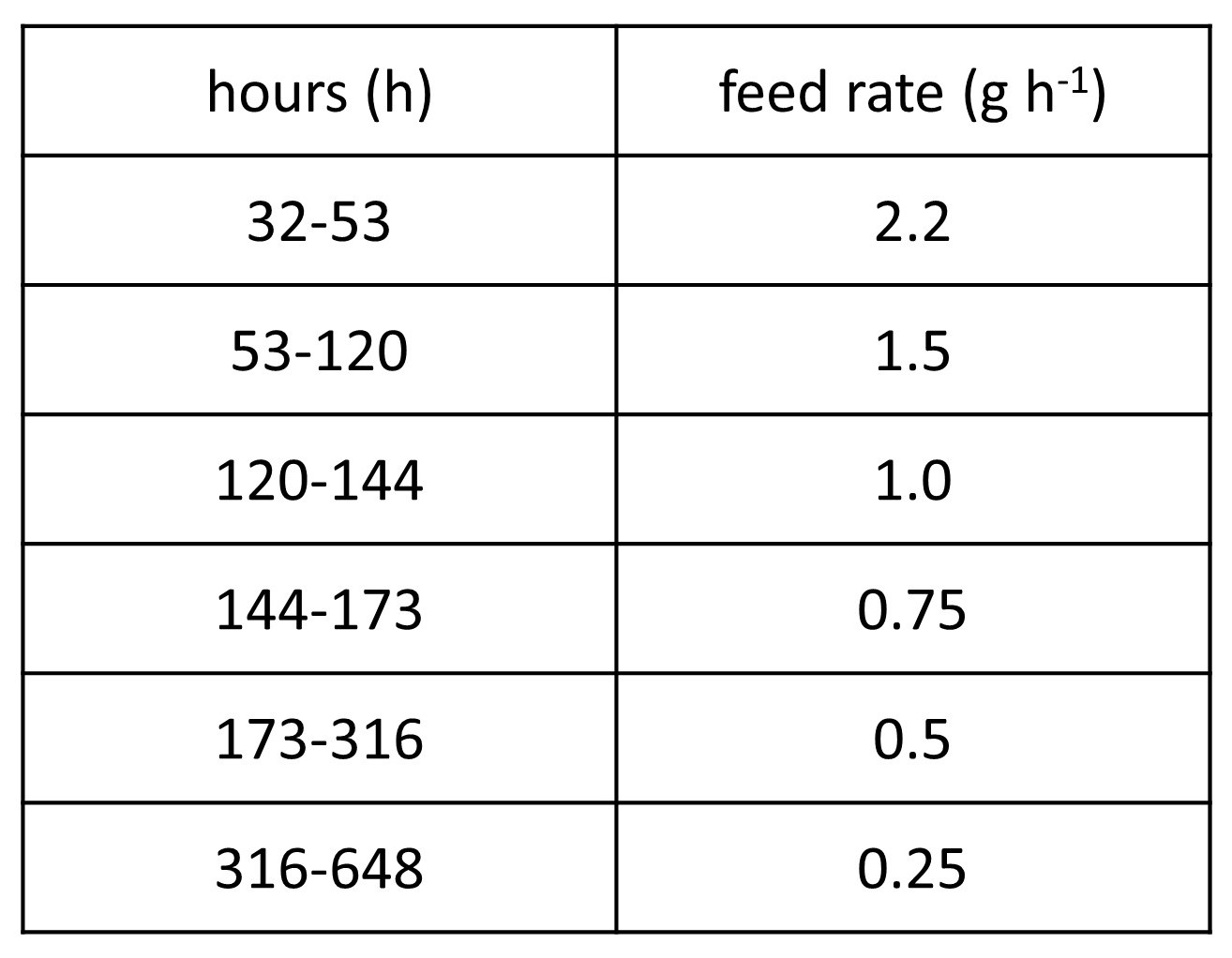

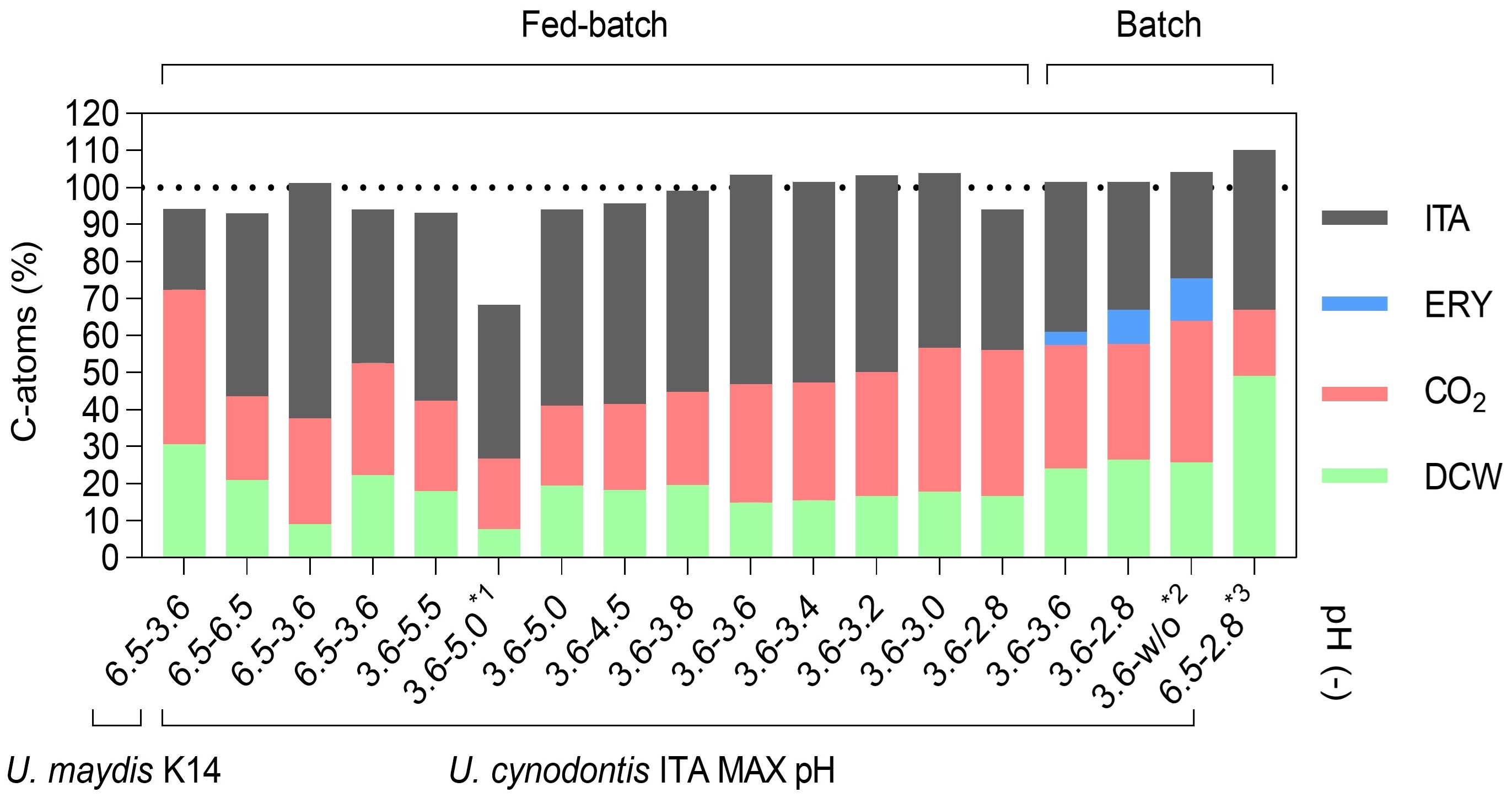


**Additional file 3: Carbon balance of all fermentations showing C-atoms in % of ITA, erythritol (ERY), CO_2_, and DCW.**

*1: with a prolonged feeding phase. *2: without pH control after the growth phase; final pH value of 2.1. *3: with thick juice as a sole carbon source.


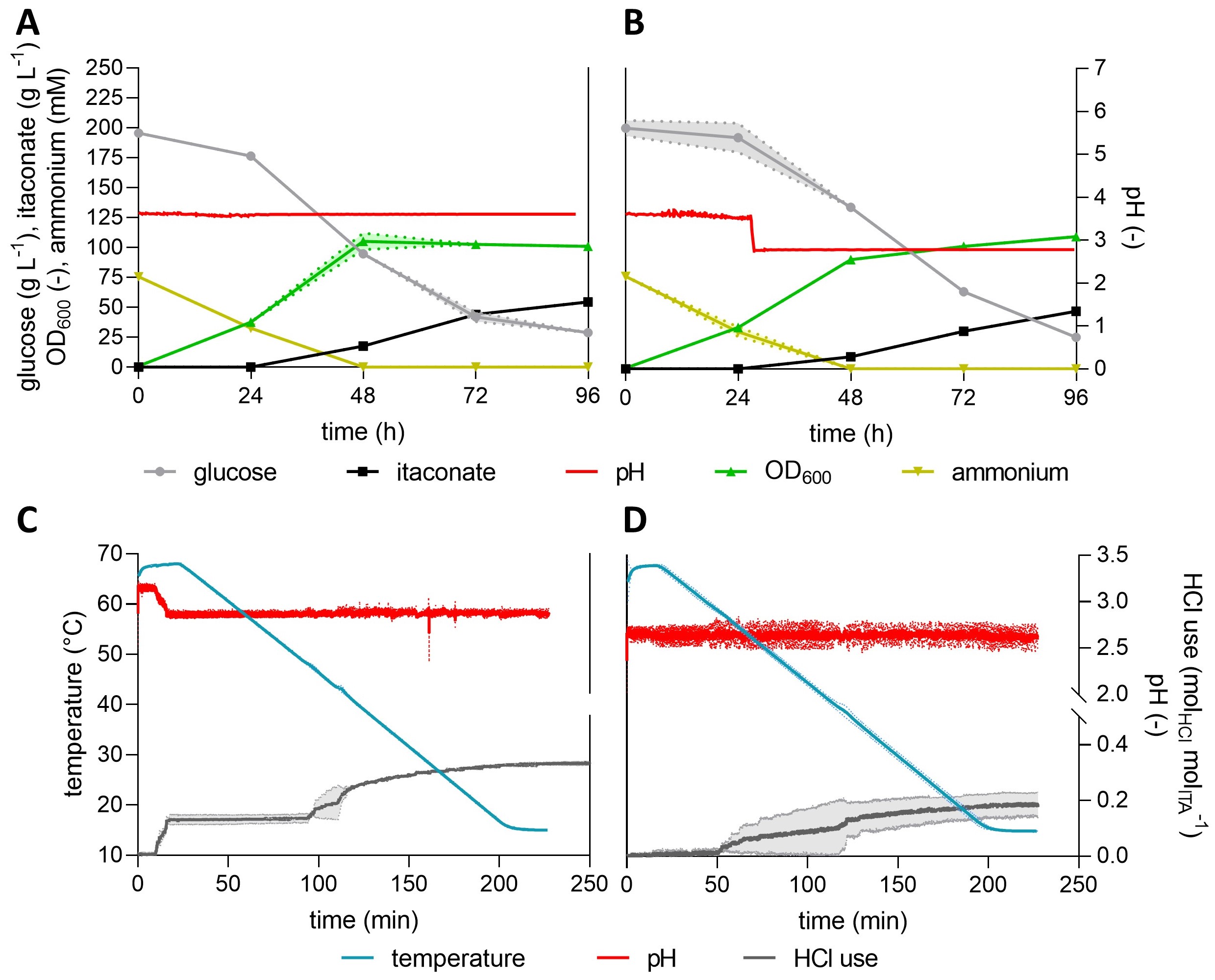


**Additional file 4: Crystallization of itaconate from batch fermentations conducted at pH 3.6 and pH 2.8.**

(A, B) concentration of glucose (●), itaconate (■), pH (red line), OD_600_ (▲) and ammonium (▼) and (C, D) temperature (blue line), pH (red line) and HCl use (grey line). High-density batch fermentations of *U. cynodontis* ITA MAX pH were performed in a bioreactor containing batch medium with approximately 200 g L^-1^ glucose and 75 mM NH_4_Cl. During the fermentation at pH 3.6, the pH was maintained at a constant level throughout the entire fermentation process (A). For the fermentation at pH 2.8, the pH was controlled at 3.6 for approximately 30 h and was then allowed to naturally decrease to 2.8 (B). Following fermentation, the cell-free broths with pH 3.6 (C) and pH 2.8 (D) were concentrated by rotary evaporation using an IKA® RV10 auto Hb rotary evaporator and an IKA® VACSTAR digital vacuum pump (IKA® Werke GmbH & Co. KG, Staufen, Germany) at 60-65 °C and 100 mbar. Afterwards, the crystallization was performed with an EasyMax 102 Titration calorimeter (Mettler Toledo, Columbus, OH, USA) equipped with a SIMDOS® O2 FEM 1.02 S pump (KNF Holding AG, Sursee, Switzerland) and an InLab® Semi Micro pH electrode (Mettler Toledo, Columbus, OH, USA). The cooling rate of 0.3 K starting from 68 °C to reach 15 °C was applied and the pH was controlled at 2.8 by addition of 5 M HCl. Crystals were filtered from mother liquor with a Whatman Grade 50 Thin filter (Cytiva Europa GmbH, Freiburg im Breisgau, Germany) and afterwards dried in a VT 6060 VACUTHERM vacuum oven (Fisher Scientific GmbH, Schwerte, Germany) at 200 mbar vacuum and 40 °C for 72 h. (A, B) The mean values with standard deviation of two independent biological replicates are shown. (C, D) The mean values with deviation of two technical replicates are shown.


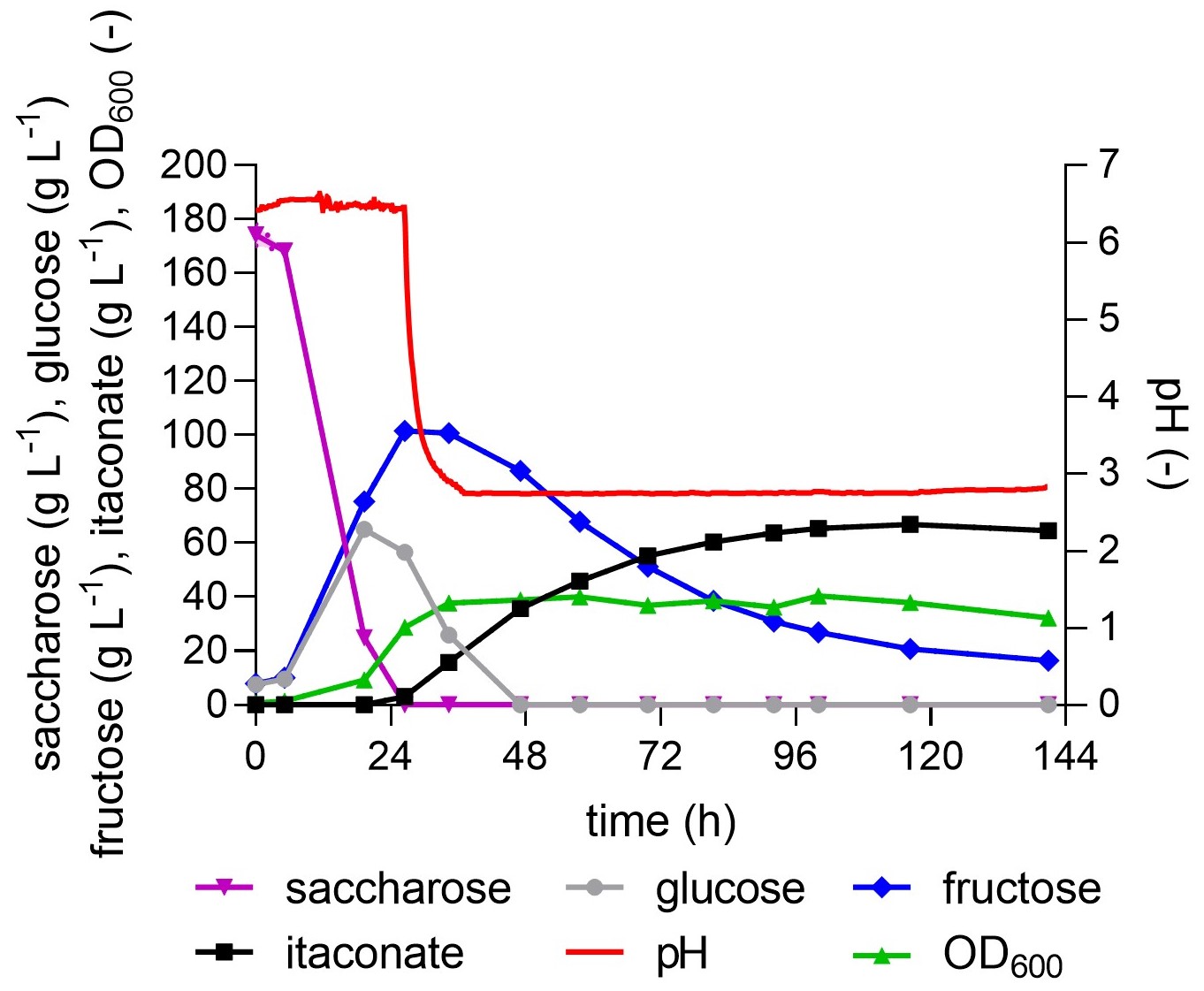


**Additional file 5: High-density batch fermentation of *U. cynodontis* ITA MAX pH with thick juice as a sole carbon source.**

Concentration of saccharose (▼), glucose (●), fructose (♦), itaconate (■), pH (red line) and OD_600_ (▲) during fermentation in a bioreactor containing batch medium with approximately 200 g L^-1^ sucrose from thick juice and 75 mM NH_4_Cl. The pH was controlled by automatic titration with 5 M NaOH. After approximately 28 h, the pH was allowed to naturally drop from pH 6.5 to pH 2.8. Upon reaching this highly acidic pH value, itaconate production continued up to a titer of approximately 65 g L^-1^ at a rate of 0.46 g L^-1^ h^-1^ and a yield of 0.4 g g^-1^. These values are higher than the ones achieved on pure glucose. This might be due to the fact that thick juice includes some other compounds such as amino acids resulting in fitter biomass and a higher carbon load. The mean values with deviation of four technical replicates of a single representative culture are shown.


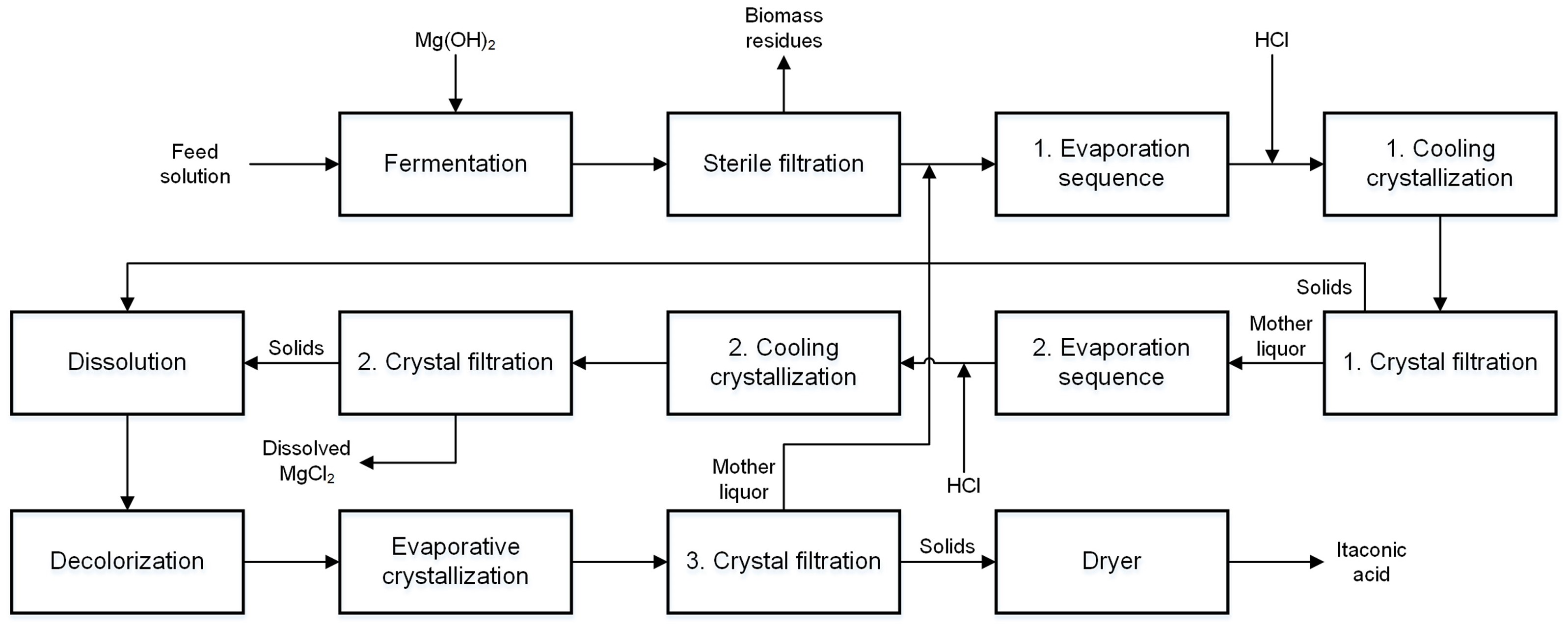


**Additional file 6: Block flow diagram of multiple crystallization process (simplified from Saur et al., 2023).**
